# Perinatal Exposure to Traffic-Related Air Pollution and Autism Spectrum Disorders

**DOI:** 10.1289/EHP118

**Published:** 2016-08-05

**Authors:** Tong Gong, Christina Dalman, Susanne Wicks, Henrik Dal, Cecilia Magnusson, Cecilia Lundholm, Catarina Almqvist, Göran Pershagen

**Affiliations:** 1 Department of Medical Epidemiology and Biostatistics, and; 2 Department of Public Health Sciences, Karolinska Institutet, Stockholm, Sweden; 3 Center for Epidemiology and Community Medicine, Stockholm County Council, Stockholm, Sweden; 4 Astrid Lindgren Children’s Hospital, Karolinska University Hospital, Stockholm, Sweden; 5 Institute of Environmental Medicine, Karolinska Institutet, Stockholm, Sweden; 6 Center for Occupational and Environmental Medicine, Stockholm County Council, Stockholm, Sweden

## Abstract

**Background:**

Studies from the United States indicate that exposure to air pollution in early life is associated with autism spectrum disorders (ASD) in children, but the evidence is not consistent with European data.

**Objective:**

We aimed to investigate the association between exposure to air pollution from road traffic and the risk of ASD in children, with careful adjustment for socioeconomic and other confounders.

**Method:**

Children born and residing in Stockholm, Sweden, during 1993–2007 with an ASD diagnosis were identified through multiple health registers and classified as cases (n = 5,136). A randomly selected sample of 18,237 children from the same study base constituted controls. Levels of nitrogen oxides (NOx) and particulate matter with diameter ≤ 10 μm (PM10) from road traffic were estimated at residential addresses during mother’s pregnancy and the child’s first year of life by dispersion models. Odds ratios (OR) and 95% confidence intervals (CI) for ASD with or without intellectual disability (ID) were estimated using logistic regression models after conditioning on municipality and calendar year of birth as well as adjustment for potential confounders.

**Result:**

Air pollution exposure during the prenatal period was not associated with ASD overall (OR = 1.00; 95% CI: 0.86, 1.15 per 10-μg/m3 increase in PM10 and OR = 1.02; 95% CI: 0.94, 1.10 per 20-μg/m3 increase in NOx during mother’s pregnancy). Similar results were seen for exposure during the first year of life, and for ASD in combination with ID. An inverse association between air pollution exposure and ASD risk was observed among children of mothers who moved to a new residence during pregnancy.

**Conclusion:**

Early-life exposure to low levels of NOx and PM10 from road traffic does not appear to increase the risk of ASD.

**Citation:**

Gong T, Dalman C, Wicks S, Dal H, Magnusson C, Lundholm C, Almqvist C, Pershagen G. 2017. Perinatal exposure to traffic-related air pollution and autism spectrum disorders. Environ Health Perspect 125:119–126; http://dx.doi.org/10.1289/EHP118

## Introduction

Autism spectrum disorders (ASD) are a range of childhood neurodevelopmental disorders characterized by deficits in communication and social interaction as well as restricted repetitive behaviors (American Psychiatric Association 2013). The global prevalence appears to have increased over the past decades (Baird et al. 2006; CDC 2014; Lauritsen et al. 2004; Nassar et al. 2009), partially due to the increased awareness and changes of clinical practices. In Sweden, 1.5% of children are currently diagnosed with ASD compared with 0.8% a decade ago (Idring et al. 2015; Lundström et al. 2015). Among ASD-affected children, intellectual disability (ID) is relatively common, and those children often display significant clinical impairment (Buescher et al. 2014).

Early twin and family studies suggested ASD to be highly heritable (Bailey et al. 1995; Lichtenstein et al. 2010; Ritvo et al. 1989); however, recent studies have shown a moderate heritability for ASD (Ronald et al. 2011; Sandin et al. 2014), suggesting a substantial nongenetic component contributing to the etiology of the disorder. Moreover, twin studies have recently reported a modest genetic component but a significant environmental contribution to the correlation between ASD and ID (Hoekstra et al. 2009, 2010). Some maternal factors such as infections during pregnancy and a history of depression or antidepressant use have been associated with ASD (Lee et al. 2015; Rai et al. 2013). There is also growing evidence that perinatal and neonatal risk factors, such as small for gestational age, preterm birth, low birth weight, and cesarean delivery, may affect the development of ASD (Buchmayer et al. 2009; Hultman et al. 2002). However, a recent meta-analysis did not indicate any specific pregnancy or delivery condition explaining the etiology of autism (Gardener et al. 2011).

Exposure to various air pollutants including ozone (Becerra et al. 2013; Jung et al. 2013; Volk et al. 2013), carbon monoxide (Becerra et al. 2013; Jung et al. 2013), nitrogen oxides (NO_x_) (Becerra et al. 2013; Jung et al. 2013; Volk et al. 2013), sulfur dioxide (Jung et al. 2013), particulate matter (PM) (Becerra et al. 2013; Jung et al. 2013; Kalkbrenner et al. 2010, 2015; Raz et al. 2015; Windham et al. 2006; Volk et al. 2013), metals (Kalkbrenner et al. 2010; Palmer et al. 2009; Windham et al. 2006), and other hazardous air pollutants (Kalkbrenner et al. 2010; Windham et al. 2006) have been linked to a modestly increased risk of ASD, which may be explained by systemic inflammation affecting the central nervous system development *in utero* (Allen et al. 2014). However, a recent European meta-analysis could not confirm this from those exposed to NO_x_ and PM during perinatal life (Guxens et al. 2016). One explanation of the discrepant findings could be residual confounding from socioeconomic factors (Braveman et al. 2005; Hajat et al. 2013; Magnusson et al. 2012; Rai et al. 2012; Thomas et al. 2012). For example, a previous register-based study in Sweden found that lower familial socioeconomic status (SES) was associated with an increased risk of ASD through multiple dimensions of SES measures (Rai et al. 2012), which is contrary to findings from the United States (Thomas et al. 2012). Pollutant-specific effects could also contribute to the discrepant findings. In one study, the positive association was found for prenatal exposure to particulate matter with an aerodynamic diameter ≤ 2.5 μm (PM_2.5_) but not to particulate matter with a diameter of ≤ 10 μm (PM_10_) (Raz et al. 2015). Additionally, associations may differ for ASD with or without accompanying intellectual impairment, although this was not seen in a recent U.S. study (Kalkbrenner et al. 2015).

Our aim was to investigate the association between exposure to air pollution from road traffic and the risk of ASD in children with or without presence of ID, with careful adjustment for individual- and area-level SES as well as other potential confounders. We selected a very large study base, allowing for more informative subgroup analyses than in earlier studies.

## Methods

### Population and Study Design

We conducted a case–control study based on the Stockholm Youth Cohort (SYC), a prospective cohort study including all children who resided in Stockholm County for at least 4 years during 2001–2007 (Idring et al. 2012). To investigate the effect of air pollution during prenatal and postnatal periods, we selected a subpopulation from SYC including children born and living in Stockholm County all the time between 1993 and 2007 and with biological mothers living in Stockholm County 1 year before and 1 year after the child’s birth (*n* = 277,478). Each child born in Sweden or each immigrant staying in Sweden for at least 1 year is assigned a unique personal identity number, which enables accurate linkage from different health registers to various sociodemographic background information while maintaining individual anonymity (Ludvigsson et al. 2009).

The cases constituted children with ASD (*n* = 5,529) identified from the National Patient Register (NPR), the Clinical Database for Child and Adolescent Psychiatry in Stockholm (PASTILL), the Habilitation Register (HAB), and the Stockholm Regional Health Care Data warehouse (VAL) until 31 December 2011. These registers cover all public-financed health seeking pathways for ASD, with or without presence of ID, in Stockholm County during the whole study period, described in detail in a validation study (Idring et al. 2012). Diagnosis and care for ASD and ID was based on the *International Classification of Diseases 9th* or *10th Revision* (ICD-9/10 codes: 299/F81 for ASD and 317–319/F70–79 for ID), the *Diagnostic and Statistical Manual of Mental Disorders, 4th Edition* (DSM-IV code: 299 for ASD and 317–319 for ID), or any use of habilitation services followed by an ASD diagnosis. Cases were further divided into ASD with and without ID, regardless of whichever diagnosis came first. We excluded adopted children (*n* = 2, 0.04%), multiple births (*n* = 162, 3.1%), and births that were not recorded in the Medical Birth Register (*n* = 229, 4.1%).

We selected a random sample of 20,000 children from the subpopulation of SYC as controls and further excluded 420 (2.1%) who developed ASD during follow-up. Among the remaining 19,580 controls, we excluded adopted children (*n* = 15, 0.1%), multiple births (*n* = 585, 3.1%), and births that were not recorded in the Medical Birth Register (*n* = 743, 3.8%).

### Exposure Assessment

Detailed descriptions of the air pollution exposure assessment methodology are available in previous publications (Bellander et al. 2001; Gruzieva et al. 2012). Briefly, a Gaussian air quality dispersion model was used to estimate the temporal and spatial distribution of NO_x_ and PM_10_ in Stockholm County during the study period. This was based on emission databases for NO_x_ in 1990, 1995, 2000, 2002–2004, and interpolations of estimated levels during the remaining years of the exposure period. PM_10_ did not show any time trends during this period, and model calculations were based on the year 2004. A street canyon contribution was added for the most polluted street segments in the inner city of Stockholm with multistory houses on both sides. Comparisons between model estimated and monitoring station–measured NO_x_ levels during the exposure period provided an *R* ^2^ of 0.74–0.80, indicating a moderate-to-high model performance (Johansson et al. 2008). For PM_10_ the *R* ^2^ was 0.61 (Eneroth et al. 2006). Relevant residential addresses of the study subjects were geocoded and pollutant levels emanating from road traffic were estimated at these coordinates from the dispersion models and used to calculate annual average concentrations for NO_x_ and PM_10_. NO_x_ is used as a marker for tail pipe emissions, such as fine particles, whereas PM_10_ reflects coarse particulates, mainly originating from road dust. To account for changes in exposure levels among those moving to another residence, time-weighted NO_x_ and PM_10_ concentrations related to road traffic emissions were calculated based on all registered addresses during the pregnancy and the child’s first year of life.

### Covariates

Information on child characteristics including birth year, sex, sibling order, gestational age, birth weight, congenital malformation and maternal characteristics including season of conception, smoking during pregnancy, and marital status at child birth were obtained from the Medical Birth Register (Swedish National Board of Health and Welfare 2003). Data regarding family characteristics at child birth including disposable income within household, maternal and paternal education, as well as employment of the mother and father were retrieved from the longitudinal integration database for health insurance and labor market studies, originally from Statistics Sweden (2016). Information on maternal and paternal age at child birth, municipality of the mother at child birth, and parental birth countries was retrieved from the Total Population Register (Statistics Sweden 2006). Furthermore, area-based SES characteristics at birth year were measured by a neighborhood deprivation index (Sariaslan et al. 2013). Neighborhood was defined by the small-area market statistics (SAMS), which is based on small socioeconomically homogeneous areas with an average of 1,000 residents. Information on welfare beneficiaries, unemployment, immigrants, divorce rate, income, education, residential mobility, and criminal conviction rate from Statistics Sweden were linked with each SAMS area to calculate a neighborhood deprivation index using principal component analysis (Sariaslan et al. 2013). The neighborhood deprivation index was further categorized into tertiles. Information on family history of psychiatric disorders including schizophrenia, bipolar disorders, ID, non-affective psychosis, and other diagnoses in the mental and behavioral disorders chapter from ICD-10 (F-diagnoses) was obtained through NPR, VAL, and PASTILL (see Table S1 for diagnostic codes).

### Statistical Analyses

To estimate the independence of pollutants over time, we calculated correlation coefficients for NO_x_ and PM_10_ over the pregnancy period of the mother and child’s first year of life. Sociodemographic characteristics were compared among cases and controls using the *t*-test or chi-square test. To assess the association between exposure to pollutants during each specific period and ASD in children and account for differences in diagnostic practices over time and across municipalities, we used conditional logistic regression models and conditioned on calendar year and municipality of birth (see Table S2 for detailed information on classification of municipalities). First, the two air pollution components were treated as continuous variables, and fixed exposure increments per 20 μg/m^3^ for NO_x_ and per 10 μg/m^3^ for PM_10_ were used in all models to estimate the risk of ASD overall, with and without ID. Second, pollutants were categorized into quartiles based on their distribution in each time period and the lowest quartiles served as reference group when modeling the association with ASD overall. To assess effect modification, we examined the association between continuous exposure to either pollutant and ASD overall, with and without ID by sex, sibling order, mother’s marital status, neighborhood deprivation, highest education between parents at child birth, mother’s smoking status during pregnancy, as well as residential mobility during mother’s pregnancy via inclusion of the interaction terms in the regression models. Wald tests were used to examine the statistical significance of interaction terms, using *p* < 0.05 as significance level. In sensitivity analyses, we excluded children with ASD diagnoses before 2 years of age, those born before year 2003 (when we have complete coverage on ASD diagnosis from birth from all registers), children with congenital malformation, those with either parent having any psychiatric disorders, children of foreign-born parents, children born to mothers with preeclampsia, pregestational and gestational diabetes, premature rupture of the membranes, placental abruption, children born before 37 weeks of gestation, or children born with a birth weight < 2,500 g, because they are often at greater risk of ASD. To further explore the potential spatial overadjustment, we provided estimates of the association between pollutants and ASD without conditioning on municipality of birth. We also ran a sensitivity analysis by including multiple births, for which the information on sibling order and birth weight was ambiguous (Swedish National Board of Health and Welfare. 2003).

We used a directed acyclic graph to determine potential confounders of the investigated associations (see Figure S1). A series of models were run step-wise to assess the gradual changes of risk estimates by further adjustment for potential confounders. We present odds ratios (OR) and 95% confidence intervals (CI) from crude models and models adjusted for sex, birth month, sibling order, maternal age, paternal age, mother’s marital status, parents’ birth countries, mother’s education, father’s education, mother’s employment, father’s employment, disposable income within household, and neighborhood deprivation. Statistical analyses were conducted using SAS version 9.4 (SAS Institute Inc., Cary, NC, USA).

The study protocol was reviewed and approved by the regional ethics review board in Stockholm, Sweden.

## Results

Table 1 lists characteristics of the study population. Compared with controls, ASD cases were on average 2 years younger, more likely to be boys, have a parent with < 12 years of education, being unemployed, or with psychiatric disorders. Maternal characteristics were also slightly different between cases and controls. For example, mothers of cases were less often married or cohabiting, but more often smoked during pregnancy.

**Table 1:** Characteristics of 5,136 ASD and 18,237 randomly selected control subjects born in Stockholm, Sweden, between 1993 and 2007.

Characteristic	Cases (*n* = 5,136)	Controls (*n* = 18,237)	*p*-Value
Child characteristics
Age at end of follow-up (years)	13.0 ± 4.1	11.5 ± 4.5	< 0.0001
Sex
Male	3,760 (73.2)	9,408 (51.6)	< 0.0001
Female	1,376 (26.8)	8,829 (48.4)
Birth year
1993–1995	1,321 (25.7)	3,527 (19.3)	< 0.0001
1996–1998	1,184 (23.1)	3,050 (16.7)
1999–2001	1,088 (21.2)	3,200 (17.5)
2002–2004	920 (17.9)	4,014 (22.0)
2005–2007	623 (12.1)	4,446 (24.4)
Sibling order
First child	2,402 (46.8)	7,785 (42.7)	< 0.0001
Not first child	2,734 (53.2)	10,452 (57.3)
Birth weight (g)
< 2,500	259 (5.0)	487 (2.6)	< 0.0001
2,500–3,000	576 (11.2)	1,777 (9.7)
3,001–3,500	1,507 (29.3)	5,845 (32.1)
3,501–4,000	1,742 (33.9)	6,561 (36.0)
> 4000	1,015 (19.8)	3,488 (19.1)
Congenital malformations
No	4,851 (94.7)	16,565 (90.8)	< 0.0001
Yes	274 (5.3)	530 (2.9)
Intellectual disability
No	4,223 (82.2)	18,119 (99.4)	< 0.0001
Yes	913 (17.8)	118 (0.6)
Family characteristics
Maternal age at child birth (years)	30.5 ± 5.3	30.9 ± 4.9	< 0.0001
Paternal age at child birth (years)	33.3 ± 6.6	33.6 ± 6.1	< 0.0001
Season of conception
Spring (March–May)	1,300 (25.3)	5,008 (27.5)	0.0042
Summer (June–August)	1,340 (26.1)	4,784 (26.2)
Autumn (September–November)	1,288 (25.1)	4,230 (23.2)
Winter (December–February)	1,208 (23.5)	4,215 (23.1)
Residential mobility during pregnancy			0.003
Nonmovers	4,025 (78.6)	14,646 (80.5)
Movers	1,095 (21.4)	3,551 (19.5)
Parents’ birth countries
Both from Sweden	3,512 (68.4)	12,796 (70.2)	0.0013
One from other countries	889 (17.3)	2,780 (15.2)
Both from other countries	713 (13.9)	2,610 (14.1)
Disposable income within household at child birth (quintiles)
Lowest	586 (11.4)	1,860 (10.2)	< 0.0001
Lower middle	1,193 (23.2)	3,509 (19.2)
Middle	1,266 (24.7)	4,229 (23.2)
Upper middle	1,142 (22.2)	4,187 (23.0)
Highest	949 (18.5)	4,452 (24.4)
Neighborhood deprivation index at child birth	0.15 ± 1.12	–0.02 ± 1.12
Low (–7.78 to –0.55)	1,463 (28.5)	6,249 (34.3)
Medium (–0.55 to 0.19)	1,643 (32.0)	6,301 (34.6)	< 0.0001
High (0.19 to 4.06)	2,030 (39.5)	5,687 (31.2)
Mother’s education at child birth
Low (≤ 9 years)	808 (15.7)	2,230 (12.2)	< 0.0001
Medium (10–12 years)	2,394 (46.6)	7,879 (43.2)
High (> 12 years)	1,921 (37.4)	8,076 (44.3)
Father’s education at child birth
Low (≤ 9 years)	856 (16.7)	2,486 (13.6)	< 0.0001
Medium (10–12 years)	2,316 (45.1)	7,781 (42.7)
High (> 12 years)	1,890 (36.8)	7,766 (42.6)
Highest education in family at child birth
At least one parent having > 12 years of education	2,571 (50.1)	10,442 (57.3)	< 0.0001
Neither parents having 12 years of education	2,564 (49.9)	7,789 (42.7)
Mother’s employment during pregnancy
Employed	3,512 (68.4)	13,888 (76.2)	< 0.0001
Unemployed with tasks	602 (11.7)	1,549 (8.5)
Unemployed without tasks	1,020 (19.9)	2,799 (15.4)
Father’s employment during pregnancy
Employed	4,200 (81.8)	15,751 (86.4)	< 0.0001
Unemployed with tasks	391 (7.6)	1,019 (5.6)
Unemployed without tasks	436 (8.5)	1,216 (6.7)
Mother’s marital status at child birth
Married/cohabiting	4,289 (83.5)	16,072 (88.1)	< 0.0001
Single/other situations	830 (16.2)	2,128 (11.7)
Parental psychiatric history (F-diagnoses)
Father diagnosed	578 (11.3)	1,925 (10.6)	< 0.0001
Mother diagnosed	1,622 (31.6)	4,266 (23.4)
Both parents diagnosed	868 (16.9)	1,552 (8.5)
Parental history of schizophrenia
Father diagnosed	18 (0.4)	25 (0.1)	0.0063
Mother diagnosed	9 (0.2)	28 (0.2)
Parental history of bipolar disorders
Father diagnosed	43 (0.8)	90 (0.5)	< 0.0001
Mother diagnosed	98 (1.9)	170 (0.9)
Parental history of intellectual disability
Father diagnosed	9 (0.2)	4 (0.0)	< 0.0001
Mother diagnosed	20 (0.4)	14 (0.1)
Parental history of non-affective psychosis
Father diagnosed	83 (1.6)	146 (0.8)	< 0.0001
Mother diagnosed	39 (0.8)	136 (0.8)
Gestational age (weeks)
< 33	79 (1.5)	129 (0.7)	< 0.0001
33–36	270 (5.3)	661 (3.6)
37–42	4,370 (85.1)	16,028 (87.9)
> 42	406 (7.9)	1,393 (7.6)
Preeclampsia
No	4,949 (96.4)	17,781 (97.5)	< 0.0001
Yes	187 (3.6)	456 (2.5)
Pregestational and gestational diabetes
No	5,037 (98.1)	18,072 (99.1)	< 0.0001
Yes	99 (1.9)	165 (0.9)
Placental abruption
No	5,107 (99.4)	18,172 (99.6)	0.0373
Yes	29 (0.6)	65 (0.4)
Premature rupture of the membranes
No	5,033 (98.0)	18,009 (98.8)	< 0.0001
Yes	103 (2.0)	228 (1.3)
Maternal smoking during pregnancy
No	3,910 (76.1)	14,476 (79.4)	< 0.0001
1–10 cigarettes/day	383 (7.5)	1,075 (5.9)
> 10 cigarettes/day	241 (4.7)	465 (2.6)
Missing	602 (11.7)	2,221 (12.2)
Note: Values are *n* (%) or mean ± SD. Missing with < 2% was not presented.			

**Figure 1:**
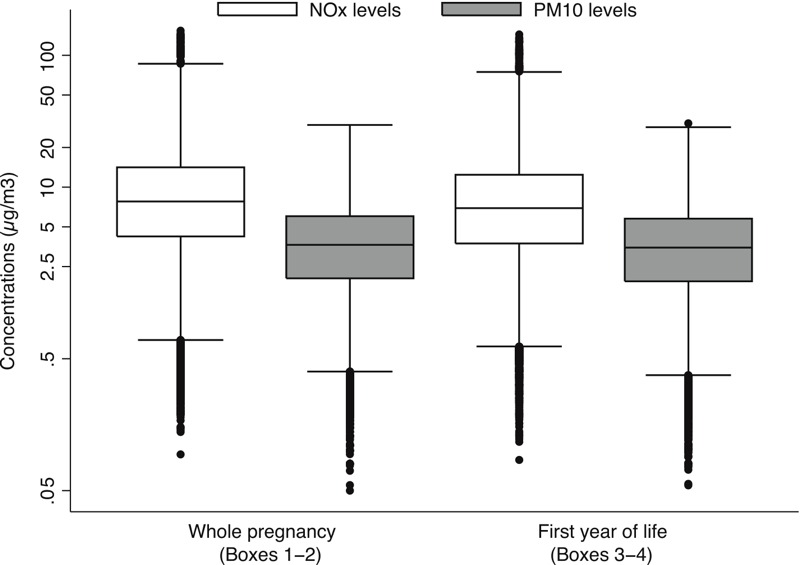
Box plot describing the distribution of NO_x_ (white) and PM_10_ (gray) concentrations (μg/m^3^) from local traffic in study population during mother’s pregnancy and first year after child birth. The box and whiskers denote the 5th, 25th, 50th, 75th, 95th percentile and outlier values of pollutants’ distributions.

Figure 1 shows box plots of air pollutant levels during the pregnancy and the child’s first year of life, respectively. The arithmetic mean levels of NO_x_ from local traffic were 11.0 μg/m^3^ during mother’s pregnancy, and dropped somewhat to 9.8 μg/m^3^ during the postnatal period (see Table S3). On the other hand, the yearly arithmetic mean levels of PM_10_ were relatively constant (4.2–4.4 μg/m^3^). NO_x_ was closely correlated with PM_10_ (*r* ^2^ ≥ 0.7) over the study period as both have local traffic as the major source.

**Figure 2:**
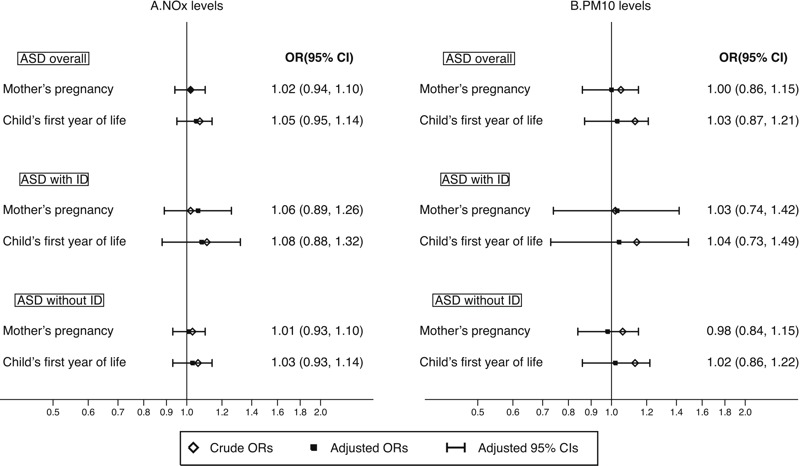
Odds ratios and 95% confidence intervals for ASD overall, ASD with or without ID by residential address-based (*A*) NO_x_ (per 10-μg/m^3^ increase) and (*B*) PM_10_ (per 20-μg/m^3^ increase) levels during mother’s pregnancy and child’s first year of life. All models were conditioned on calendar year of birth and municipality of birth, as well as adjusted for sex, birth month, sibling order, maternal age, paternal age, mother’s marital status, parents’ birth countries, mother’s education, father’s education, mother’s employment, father’s employment, disposable income within household, and neighborhood deprivation.

The associations between air pollution exposure as a continuous measure and the risk of ASD overall, with and without ID, are shown in Figure 2. We did not observe any differences in risk of ASD overall, with or without ID, by exposure during pregnancy to NO_x_ or PM_10_ after adjusting for potential confounders [e.g., adjusted OR was 1.02 (95% CI: 0.94, 1.10) for ASD overall by per 20-μg/m^3^ increase of NO_x_ and 1.06 (95% CI: 0.89, 1.26) for ASD with ID, and 1.01 (95% CI: 0.93, 1.10) for ASD without ID]. Corresponding ORs for an increment of 10 μg/m^3^ of PM_10_ were 1.00 (95% CI: 0.86, 1.15), 1.03 (95% CI: 0.74, 1.42), and 0.98 (95% CI: 0.84, 1.15), respectively. Results were similar for exposure during the child’s first year of life (Figure 2; see also Table S4). However, when pollutants were categorized into quartiles, children living in areas within the 3rd and 4th quartiles of air pollution exposure had a slightly lower risk of ASD overall compared with those living in areas within the least polluted quartile, especially in relation to exposure during pregnancy (Table 2). We observed no major confounding by individual or neighborhood covariates on the association between pollutant levels and ASD (Table 2; see also Table S4). However, the adjustments consistently generated somewhat lower ORs for ASD without ID, which constituted the major group among the cases.

**Table 2:** Risk of ASD overall by quartiles of modeled pollutants’ levels at different time windows among 5,136 ASD cases and 18,237 controls born in Stockholm, Sweden, between 1993 and 2007.

Time windows and pollutants	No. of cases/controls	Models A^*a*^ OR (95% CI)	Models B^*b*^ OR (95% CI)	Models C^*c*^ OR (95% CI)
Entire pregnancy	5,112/18,192
NO_x_
2nd quartile	1,340/4,486	1.01 (0.91, 1.13)	0.98 (0.88, 1.10)	0.96 (0.85, 1.07)
3rd quartile	1,285/4,541	0.94 (0.83, 1.06)	0.91 (0.80, 1.03)	0.88 (0.78, 1.00)
4th quartile	1,279/4,547	0.94 (0.82, 1.09)	0.91 (0.78, 1.05)	0.89 (0.77, 1.03)
PM_10_
2nd quartile	1,399/4,427	1.03 (0.92, 1.14)	1.01 (0.91, 1.12)	0.98 (0.88, 1.09)
3rd quartile	1,247/4,579	0.92 (0.81, 1.04)	0.90 (0.79, 1.02)	0.87 (0.77, 0.99)
4th quartile	1,140/4,686	0.93 (0.81, 1.06)	0.88 (0.77, 1.02)	0.86 (0.75, 0.99)
First year of life	5,121/18,225
NO_x_
2nd quartile	1,321/4,515	1.05 (0.94, 1.18)	1.02 (0.91, 1.15)	0.98 (0.87, 1.10)
3rd quartile	1,328/4,509	1.04 (0.91, 1.19)	1.00 (0.87, 1.14)	0.95 (0.83, 1.09)
4th quartile	1,297/4,539	1.06 (0.90, 1.25)	1.00 (0.85, 1.18)	0.96 (0.81, 1.13)
PM_10_
2nd quartile	1,398/4,439	1.10 (0.99, 1.23)	1.08 (0.97, 1.21)	1.03 (0.92, 1.16)
3rd quartile	1,280/4,557	1.03 (0.90, 1.17)	0.98 (0.86, 1.12)	0.93 (0.82, 1.07)
4th quartile	1,160/4,676	1.05 (0.90, 1.22)	0.97 (0.83, 1.13)	0.92 (0.78, 1.07)
^***a***^Models were conditioned on calendar year of birth and municipality of birth, as well as adjusted for sex and birth month. ^***b***^Models were conditioned on calendar year of birth and municipality of birth, as well as adjusted for sex, birth month, birth order, parents’ birth countries, mother’s marital status, mother’s education, father’s education, mother’s employment, father’s employment, and disposable income within household. ^***c***^Models were conditioned on calendar year of birth and municipality of birth, as well as adjusted for sex, birth month, birth order, parents’ birth countries, mother’s marital status, mother’s education, father’s education, mother’s employment, father’s employment, disposable income within household, and neighborhood deprivation.				

**Figure 3:**
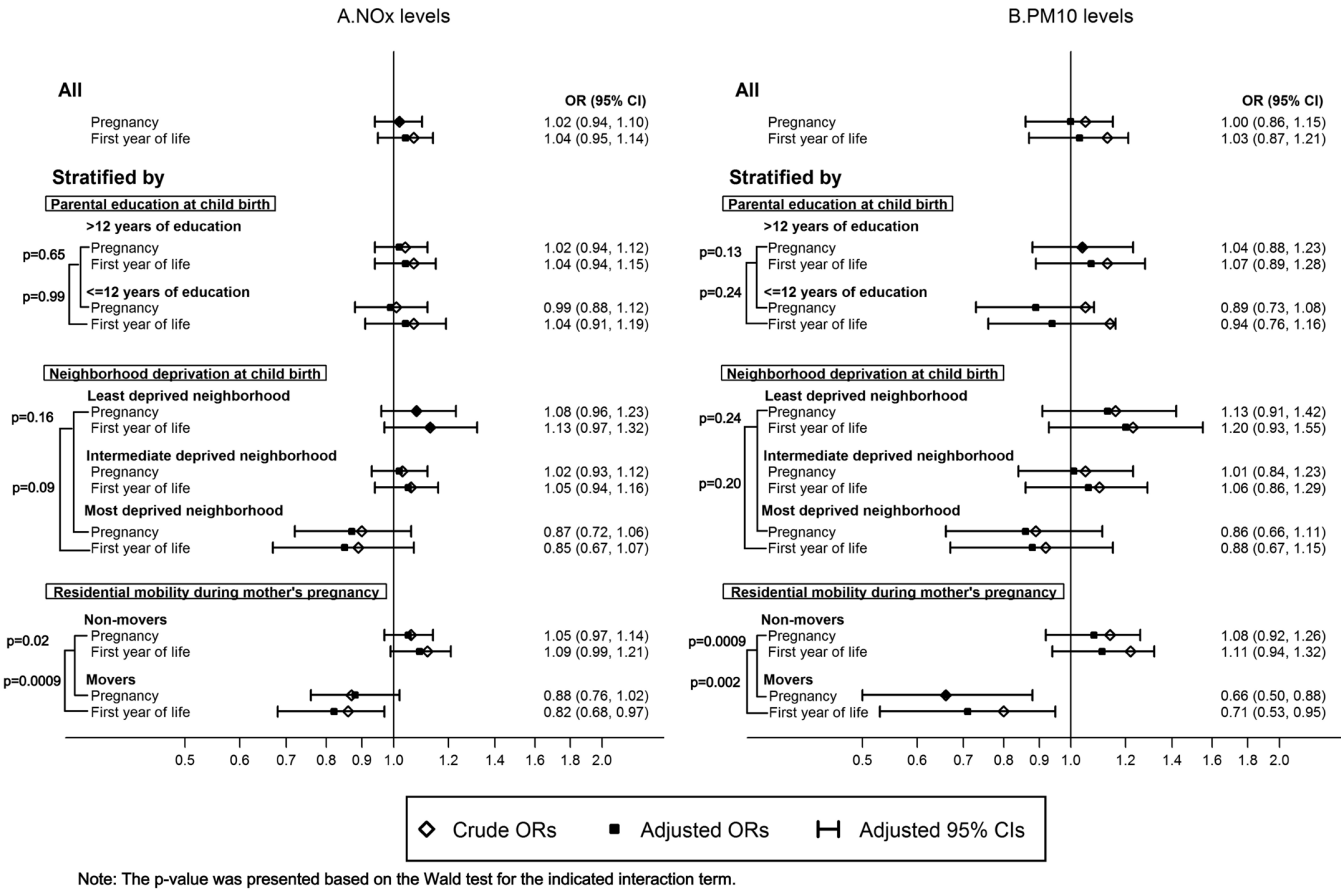
Odds ratios and 95% confidence intervals for ASD overall by residential address-based (*A*) NO_x_ (per 10-μg/m^3^ increase) and (*B*) PM_10_ (per 20-μg/m^3^ increase) levels during mother’s pregnancy and child’s first year of life in stratified samples. All models were conditioned on calendar year of birth and municipality of birth, as well as adjusted for sex, birth month, sibling order, maternal age, paternal age, mother’s marital status, parents’ birth countries, mother’s education, father’s education, mother’s employment, father’s employment, disposable income within household, and neighborhood deprivation as covariates.

We observed a similar pattern of associations in subgroups based on parental education (Figure 3 for ASD overall; see also Figures S2 and S3 for ASD with and without ID). There was a suggested inverse relation between air pollution exposure and ASD for those in the most deprived neighborhoods, especially for ASD without ID. A statistically significant interaction was seen for residential mobility, where a decreased risk for ASD with air pollution exposure pre- or postnatally was seen only among those changing residential address during pregnancy (all *p*-interactions < 0.03). Further analyses showed that moving patterns were related to SES and psychiatric illness among the parents (see Table S5). Interactions with air pollution exposure in relation to ASD were also investigated for several other characteristics, including sex, sibling order, mother’s smoking and marital status, but no statistically significant effect modification was observed (data not shown).

In sensitivity analyses including multiple births (see Table S6) or excluding children born to mothers with changing residential address during pregnancy, cases diagnosed before 2 years of age, children with congenital malformation, children born before 2003, children born to mothers with preeclampsia, pregestational and gestational diabetes, premature rupture of the membranes, placental abruption, children of foreign-born parents, children born at < 37 weeks gestation or with a birth weight < 2,500 g, we found similar patterns of results for both pollutants as in our main analysis (see Table S7). Furthermore, in analyses not conditioning on municipality of birth, we observed that exposure to NO_x_ and PM_10_ during pre- or postnatal period appeared to be associated with a decreased risk of ASD (see Table S8).

## Discussion

Among children born between 1993 and 2007 in Stockholm County, we found that pre- and postnatal exposure to either NO_x_ or PM_10_ was not associated with ASD overall, with or without ID. There was an inverse association between air pollution exposure and ASD risk for children of mothers who changed residential addresses during pregnancy, which could be partly explained by confounding by SES and psychiatric diseases in the parents.

We previously reported no association between traffic-related air pollution during pre- or postnatal periods and subclinical ASD outcomes using a twin cohort from Stockholm; however, risk estimates tended to lie below one with wide confidence intervals (Gong et al. 2014). The present study showed a similar result using a much larger sample but contradicts the positive associations reported in several previous studies from the United States (Kalkbrenner et al. 2010, 2015; Raz et al. 2015; Roberts et al. 2013; Volk et al. 2011, 2013). One possible explanation for the inconsistent results could be the lower levels of air pollution in Stockholm. For example, mean levels of PM_10_ in California and Taiwan were reported at about 25–36 and 58 μg/m^3^ (Becerra et al. 2013; Jung et al. 2013; Kalkbrenner et al. 2015; Volk et al. 2013). However, the local traffic related PM_10_ concentrations in the current study was 4.3 μg/m^3^ during mother’s pregnancy and child’s first year of life, and the background PM_10_ level generated from long-distance transportation near Stockholm County has remained at a rather stable level at 10 μg/m^3^ across the whole study period (Burman and Norman 2013). Previous studies have generally reported on nitrogen dioxide (NO_2_) with mean levels in California and Taiwan of 32–43 μg/m^3^ (converted from 17 and 28.8 ppb with temperature at 25°C) (Becerra et al. 2013; Jung et al. 2013; Volk et al. 2013). We used NO_x_ as a marker because it better reflects the tail-pipe emissions, but it is less often measured or reported. The average urban background levels of NO_2_ during the study period in Stockholm decreased from around 20 to 14 μg/m^3^ (Durant et al. 2014), and were thus considerably lower than the levels in the areas reported in earlier publications. Furthermore, the air pollution levels in Stockholm County, constituting the catchment area for our cohort and another cohort included in a recent European meta-analysis, are lower than in most (NO_x_) or all (PM_10_) other areas included this meta-analysis (Guxens et al. 2016). It is possible that we may have missed an association that is primarily seen at higher levels of exposure.

Residential mobility during pre- and postnatal life could also contribute to the inconsistent results. Young maternal age, being unmarried, having psychiatric diseases, and low SES have been associated with residential mobility during pregnancy (Bell and Belanger 2012; Fell et al. 2004; Miller et al. 2010; Tulloch et al. 2010) and may thereby affect offspring’s ASD risk estimates (Rai et al. 2012; Roberts et al. 2013; Thomas et al. 2012). Most previous studies have not investigated the potential impact by residential mobility (Jung et al. 2013; Kalkbrenner et al. 2015; Roberts et al. 2013; Volk et al. 2013). One report from the Nurses’ Health Study II found that the positive association of PM_2.5_ and ASD was stronger among nonmovers, although no interaction test was performed based on moving status (Raz et al. 2015). We observed an inverse association among families that changed their residential addresses during mother’s pregnancy. The reason for this is unclear, and the influence of moving residence on the association between air pollution and ASD, which was related to SES and psychiatric disorders among the parents, should be investigated in further studies. Furthermore, misclassification of municipality at delivery among movers could contribute to the observed inverse association because we did not take into account the effect of municipalities where the mother lived before delivery.

Another explanation of the inconsistent results could be different proportions of high- and low-functioning ASD in different study settings. For example, one study in the United States reported that 40% of the ASD cases also had ID (Kalkbrenner et al. 2015), but there were only 19% of ASD cases with co-existing ID diagnosis in our study. If the positive association could only be seen in this subtype of ASD, the higher proportion of ASD without ID in our study might have resulted in diluted associations.

Residual confounding could also contribute to the inconsistent findings by socioeconomic indicators, for example. Children from higher-SES families were more likely to be diagnosed with ASD in previous studies, and the OR estimates for both air pollutants appeared slightly lower after adjusting for confounders including birth year, birth order, parental age, income, education, and ethnicity (Becerra et al. 2013; Kalkbrenner et al. 2015; Raz et al. 2015; Roberts et al. 2013; Thomas et al. 2012; Volk et al. 2013). Family and contextual SES covariates had relatively small effects on the association between air pollutants and ASD in our study. Sweden has a rather universal health care system compared with the private sector–dominated health care system in the United States (Anell et al. 2012), which may lead to differences in case ascertainment and bias related to SES.

We could not completely rule out the possibility of an association in certain subgroups, such as in those born to mothers who did not move residence during pregnancy and among children of parents without any psychiatric diagnoses. As noted above, residential mobility, SES, and psychiatric disorders were related, making it difficult to disentangle their specific influence on the associations between air pollution exposure and ASD. A familial aggregation of ASD and other psychiatric conditions has been documented (Daniels et al. 2008). Our data suggest that the effect of air pollution on ASD could be masked by confounding from psychiatric diagnoses, partly related to differential moving patterns and SES.

Our study has several strengths. First, the linkage of encrypted data from multiple registers enabled us to include cases and controls from the same study base, and to retrieve detailed information on validated outcomes (Idring et al. 2012) and highly reliable measures at individual level. Second, the air pollution exposure assessment methodology has been validated and has generated positive associations for several outcomes in children of our study area, such as asthma, allergy, and lung function disturbances (Melén et al. 2008; Nordling et al. 2008; Schultz et al. 2012). Using register-based information on moving dates for each residential address, we also considered time-weighted estimates of pollutant levels based on the duration of stay at the respective address. Third, because of the large sample size, we had sufficient statistical power to perform subgroup-specific analyses.

Our study also has some limitations. Due to the late establishment of PASTILL, HAB, and VAL registers, we did not have full age coverage of the diagnoses for all children in the study base. However, we found a similar result on the association of pollutant levels and ASD in the subpopulation where complete information on ASD diagnoses from all registers was available among children born since 2003. Furthermore, there was no data on maternal exposure before conception or on paternal exposure during the child’s early life. The analyses conditioned on municipality (and birth year) to minimize risks for differences in diagnostic practices influencing the results. This may have led to some overadjustment in relation to the air pollution effects. However, we did not observe an increased risk by exposure to either pollutant in sensitivity analysis not conditioning on municipality.

In conclusion, our results indicate that exposure to NO_x_ and PM_10_ during the pre- and postnatal period is not associated with ASD. The absence of positive associations in our study may be related to comparatively low air pollutant levels.

## Supplemental Material

(620 KB) PDF
