# Supplementary material for: Perinatal Exposure to Traffic-Related Air Pollution and Autism Spectrum Disorders
Source: Environ Health Perspect. 2016 Aug 5;125(1):119–26. doi: 10.1289/EHP118 (PMC5226686; doi:10.1289/EHP118)
Supplement: (620 KB) PDF [file EHP118.s001.acco.pdf]

Environ Health Perspect
DOI: 10.1289/EHP118

Note to readers with disabilities: *EHP* strives to ensure that all journal content is accessible to all readers. However, some figures and Supplemental Material published in *EHP* articles may not conform to 508 standards due to the complexity of the information being presented. If you need assistance accessing journal content, please contact ehp508@niehs.nih.gov. Our staff will work with you to assess and meet your accessibility needs within 3 working days.

Supplemental Material

Perinatal Exposure to Traffic-Related Air Pollution and Autism Spectrum Disorders

Tong Gong, Christina Dalman, Susanne Wicks, Henrik Dal, Cecilia Magnusson, Cecilia Lundholm, Catarina Almqvist,and Göran Pershagen

Table of contents

Figure S1.Directed acyclic graph to determine potential confounders of the investigated associations 3

Figure S2. Odds ratios and 95% confidence intervals for ASD with ID by residential address-based NO_x (per 10 µg/m^3 increase) and PM_{10} (per 20 µg/m^3 increase) levels during mother's pregnancy and child's first year of life. 5

Figure S3. Odds ratios and 95% confidence intervals for ASD without ID by residential address-based NO_x (per 10 µg/m^3 increase) and PM_{10} (per 20 µg/m^3 increase) levels during mother's pregnancy and child's first year of life. 7

Table S1. All relevant diagnostic codes used in this project from the International Classification of Diseases (ICD) and the Diagnostic and Statistical Manual of Mental Disorders (DSM). 9

Table S2. The municipality division of Stockholm County 10

Table S3. Descriptive statistics and correlation between pollutants 11

Table S4. Risk of ASD (overall, with and without ID) based on continuous pollutants' levels during different time windows 12

Table S5. Parental SES characteristics and psychiatric diagnoses among movers and non-movers 14

Table S6. Sensitivity analysis: Risk of ASD (overall, with and without ID) based on continuous pollutants' levels during different time windows including multiple births 15

Table S7. Sensitivity analyses: Association between pollutants' levels during mother's pregnancy and child's first year of life and risk of childhood ASD overall by excluding children with certain conditions related to ASD. 17

Table S8. Risk of ASD (overall, with and without ID) based on continuous pollutants' levels during different time windows without conditioning on municipality of birth 21

Figure S1.Directed acyclic graph to determine potential confounders of the investigated associations

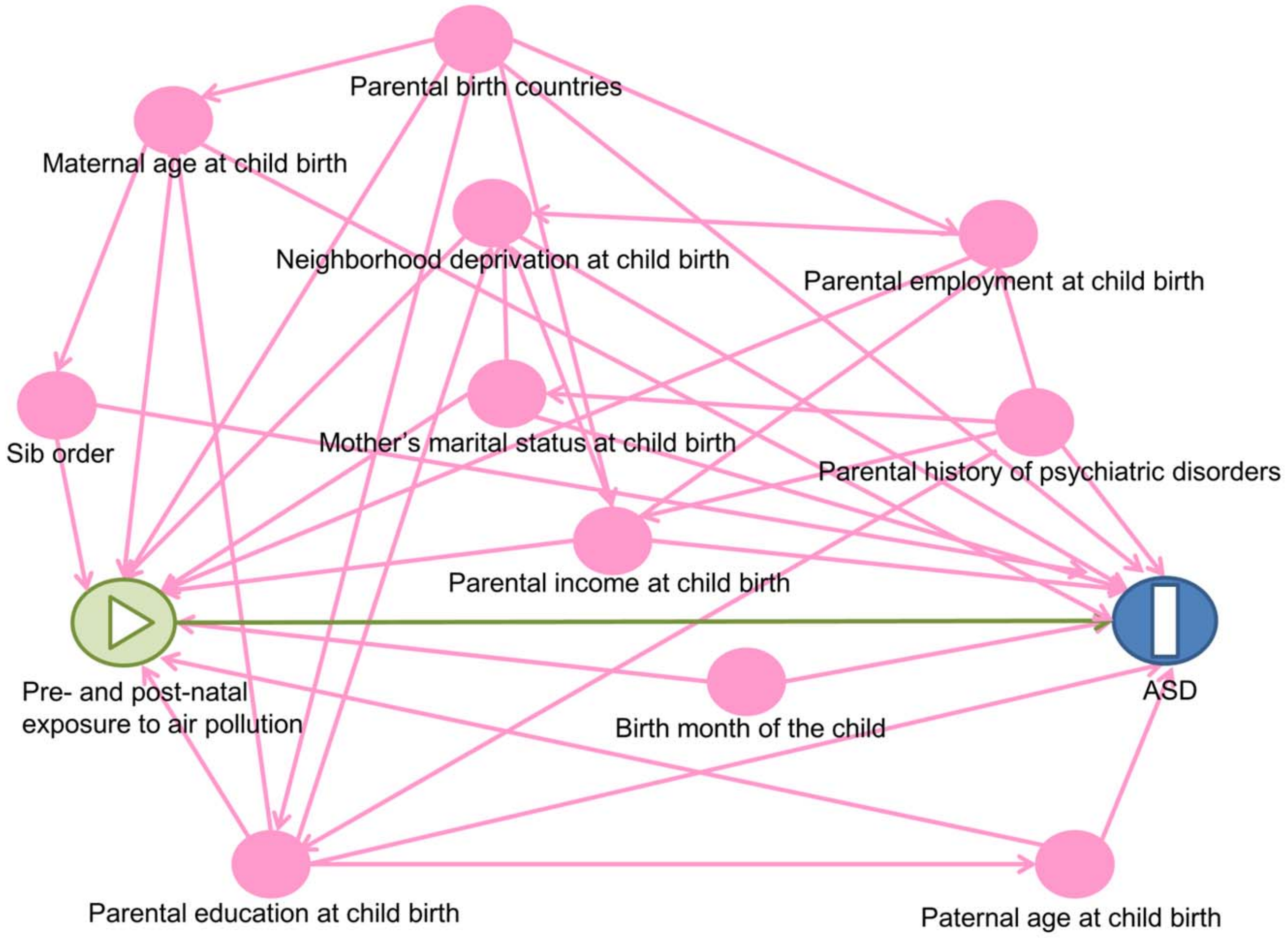

Note: We conditioned on calendar year and municipality of birth for all analyses. Therefore, they was not shown in the directed acyclic graph.

Minimal sufficient adjustment sets for estimating the total effect of pre- and post-natal exposure to air pollution on ASD: birth month of the child, maternal age at child birth, mother's marital status at child birth, neighborhood deprivation at child birth, parental birth countries, parental education at child birth, parental employment at child birth, parental income at child birth, paternal age at child birth, and sib order.

Figure S2. Odds ratios and 95% confidence intervals for ASD with ID by residential address-based NO_x (per 10 μg/m^3 increase) and PM_{10} (per 20 μg/m^3 increase) levels during mother's pregnancy and child's first year of life.

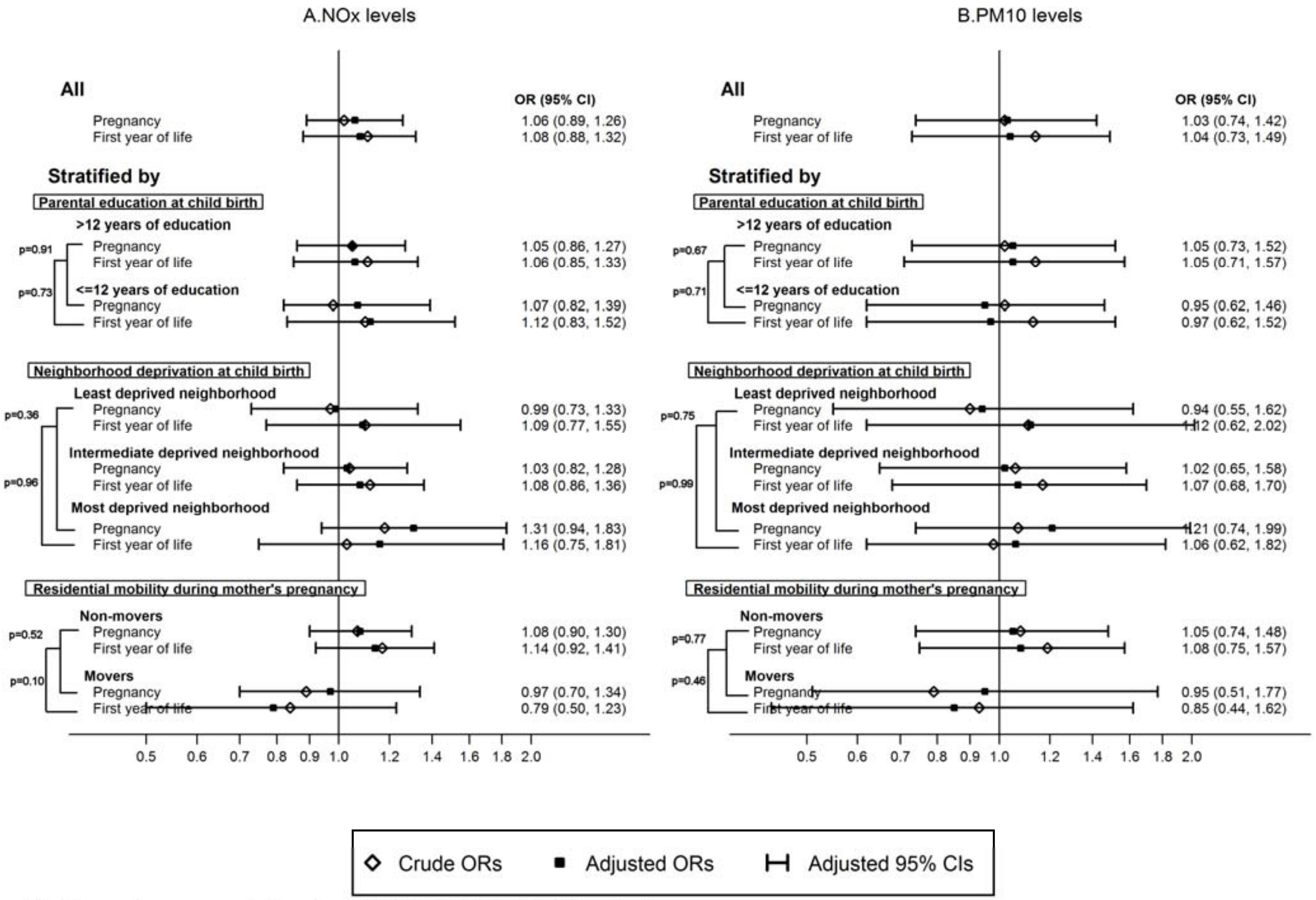

Note: The p-value was presented based on the Wald test for the indicated interaction term.

Models conditioned on calendar year and municipality of birth as well as adjusted for gender, birth month, sib order, maternal age, paternal age, mother's marital status, parents' birth countries, mother's education, father's education, mother's employment, father's employment, disposable income within household, and neighborhood deprivation.

Figure S3. Odds ratios and 95% confidence intervals for ASD without ID by residential address-based NO_x (per 10 μg/m^3 increase) and PM_{10} (per 20 μg/m^3 increase) levels during mother's pregnancy and child's first year of life.

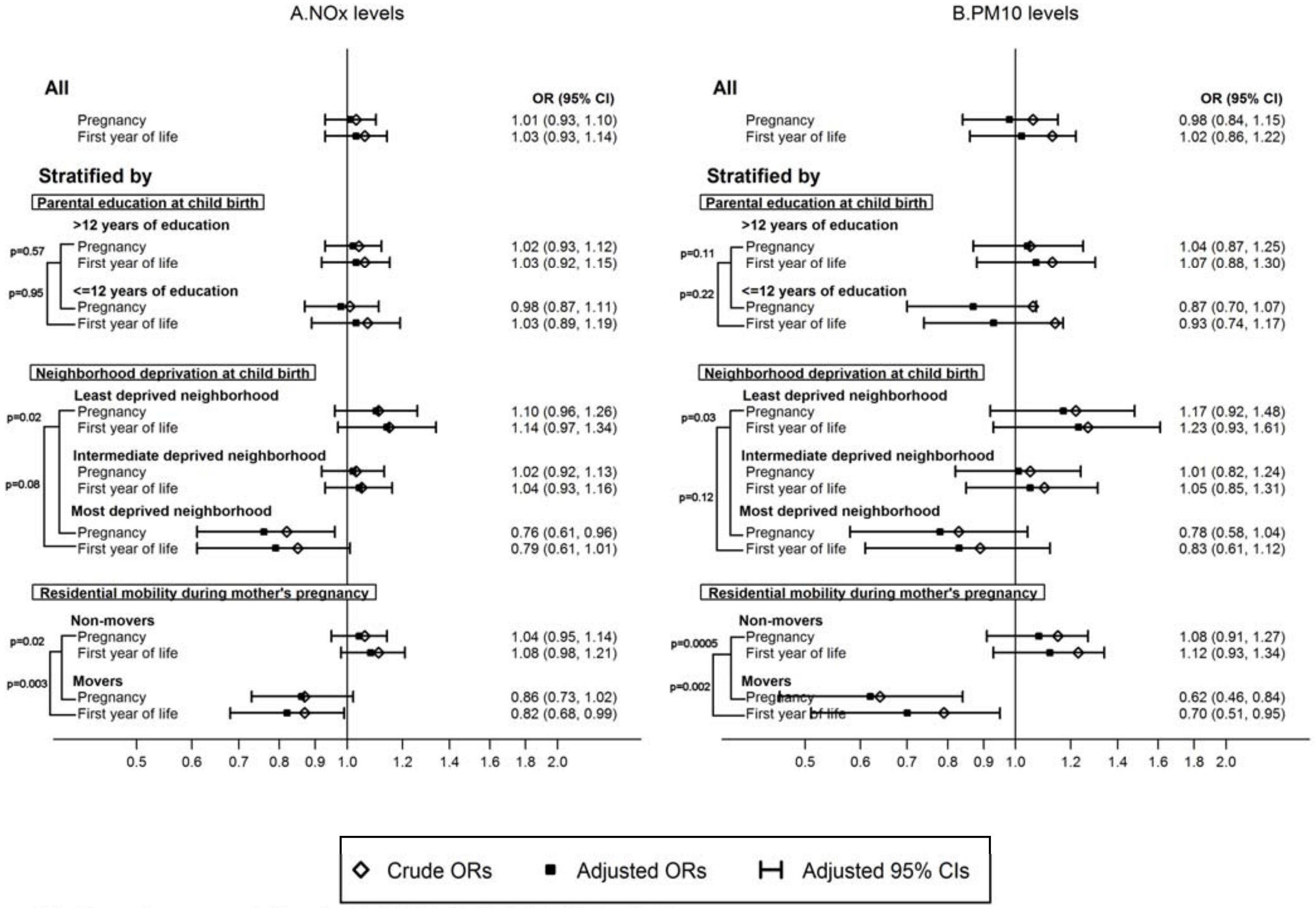

Note: The p-value was presented based on the Wald test for the indicated interaction term.

Models conditioned on calendar year and municipality of birth as well as adjusted for gender, birth month, sib order, maternal age, paternal age, mother's marital status, parents' birth countries, mother's education, father's education, mother's employment, father's employment, disposable income within household, and neighborhood deprivation.

Table S1. All relevant diagnostic codes used in this project from the International Classification of Diseases (ICD) and the Diagnostic and Statistical Manual of Mental Disorders (DSM).

Disorders	DSM-IV	ICD-10 (1997-)	ICD-9 (1987-1996)	ICD-8 (1969-1986)	ICD-7 (1957-1968)
Any psychiatric disorders	-	F	290-315	290-319	300-326
Autism spectrum disorders (ASD)	299	F84	299	N/A	N/A
Bipolar disorders	-	F30-F31	296A, C-E	29610-29630	N/A
Congenital malformations	-	Q	740-759	N/A	N/A
Intellectual disability	317-319	F70-F79	317-319	N/A	N/A
Non-affective psychosis	-	F20-29	295, 297, 298 (excl 298A and 298B)	295, 297, 298 (excl 29800 and 29810), 29999	N/A
Schizophrenia	-	F20	295 without a letter, 295A-E, G, W, X	295 excluding 29550 and 29570	N/A
Pre-eclampsia		O14-15	642E–G	N/A	N/A
Pre-gestational and gestational diabetes		E10-14, O240-243, O244	250, 648A, 648W	N/A	N/A
Premature rupture of the membranes		O42	658B	N/A	N/A
Placental abruption		O45	641C	N/A	N/A

Note: N/A= Children born/mothers delivering during1993-2007 were not in this ICD-system.

Table S2. The municipality division of Stockholm County

Stockholm County (including 26 municipalities)	
**Stockholm municipality**	**The other 25 municipalities**
Kista+Spånga+Rinkeby+Tensta	Botkyrka
Bromma	Danderyd
Häseelby+Vällingby	Ekerö
Kungsholmen+Norrmalm+Östermalm	Haninge
Södermalm	Huddinge
Skarpnäck	Järfälla
Farsta	Lidingö
Liljeholmen+Hägersten+Älvsjö+Enskede+Årsta+Vantör	Nacka
Skärholmen	Norrtälje
	Nykvarn
	Nynäshamn
	Salem
	Sigtuna
	Sollentuna
	Solna
	Sundbyberg
	Södertälje
	Tyresö
	Täby
	Upplands-Bro
	Upplands Väsby
	Vallentuna
	Vaxholm
	Värmdö
	Österåker

Table S3. Descriptive statistics and correlation between pollutants

Time	Pollutants' descriptive statistics						Pearson correlations			
	Pollutant	Mean	SD	Median	IQR	Missing n (%)	Entire pregnancy		First year after birth	
							NO_x	PM_{10}	NO_x	PM_{10}
Entire pregnancy	NO_x	11.04	11.39	7.79	9.91	69 (0.3)	1.00			
	PM_{10}	4.38	3.22	3.65	3.99		0.83	1.00		
First year after birth	NO_x	9.83	10.33	6.93	8.68	27 (0.1)	0.89	0.74	1.00	
	PM_{10}	4.21	3.14	3.48	3.05		0.74	0.89	0.83	1.00

Table S4. Risk of ASD (overall, with and without ID) based on continuous pollutants' levels during different time windows

Time windows and pollutants	Models A[†] OR (95% CI)	Models B[‡] OR (95% CI)	Models C[‖] OR (95% CI)
ASD overall (nr of cases/controls=5,136/18,237)			
Entire pregnancy			
NO_x (per 20μg/m^3 increase)	1.02 (0.95, 1.11)	1.02 (0.94, 1.10)	1.02 (0.94, 1.10)
PM_{10} (per 10μg/m^3 increase)	1.05 (0.91, 1.21)	1.01 (0.87, 1.17)	1.00 (0.86, 1.15)
First year of life			
NO_x (per 20μg/m^3 increase)	1.07 (0.97, 1.17)	1.05 (0.95, 1.15)	1.04 (0.95, 1.14)
PM_{10} (per 10μg/m^3 increase)	1.13 (0.96, 1.33)	1.06 (0.90, 1.25)	1.03 (0.87, 1.21)
ASD with ID (nr of cases/controls=913/118)			
Entire pregnancy			
NO_x (per 20μg/m^3 increase)	1.02 (0.86, 1.22)	1.06 (0.89, 1.26)	1.06 (0.89, 1.26)
PM_{10} (per 10μg/m^3 increase)	1.02 (0.75, 1.39)	1.05 (0.76, 1.45)	1.03 (0.74, 1.42)
First year of life			
NO_x (per 20μg/m^3 increase)	1.11 (0.91, 1.35)	1.09 (0.89, 1.34)	1.08 (0.88, 1.32)
PM_{10} (per 10μg/m^3 increase)	1.14 (0.81, 1.60)	1.08 (0.76, 1.54)	1.04 (0.73, 1.49)
ASD without ID (nr of cases/controls=4,223/18,119)			
Entire pregnancy			
NO_x (per 20μg/m^3 increase)	1.03 (0.95, 1.12)	1.01 (0.93, 1.10)	1.01 (0.93, 1.10)
PM_{10} (per 10μg/m^3 increase)	1.06 (0.90, 1.23)	1.00 (0.85, 1.17)	0.98 (0.84, 1.15)
First year of life			
NO_x (per 20μg/m^3 increase)	1.06 (0.96, 1.17)	1.04 (0.94, 1.15)	1.03 (0.93, 1.14)
PM_{10} (per 10μg/m^3 increase)	1.13 (0.95, 1.34)	1.05 (0.88, 1.26)	1.02 (0.86, 1.22)

† Models conditioned on calendar year and municipality of birth, as well as adjusted for gender and birth month.

‡ Models conditioned on calendar year and municipality of birth, as well as adjusted for gender, birth month, sib order, parents' birth countries, mother's marital status, mother's education, father's education, mother's employment, father's employment, and disposable income within household.

‖ Models conditioned on calendar year and municipality of birth, as well as adjusted for gender, birth month, sib order, parents' birth countries, mother's marital status, mother's education, father's education, mother's employment, father's employment, disposable income within household, and neighborhood deprivation.

Table S5. Parental SES characteristics and psychiatric diagnoses among movers and non-movers

	Non-movers (n=18,727)	Movers (n=4,646)	p
Disposable income within household at child birth (quintiles)			
Lowest	1,956 (10.5)	487 (10.5)	0.85
Lower middle	3,728 (20.0)	959 (20.6)	
Middle	4,401 (23.6)	1,081 (23.3)	
Upper middle	4,253 (22.8)	1,063 (22.9)	
Highest	4,333 (23.2)	1,056 (22.7)	
Neighborhood deprivation (tertiles)			
Least deprivation	6,269 (33.6)	1,425 (30.7)	
Intermediate deprivation	6,377 (34.1)	1,541 (33.2)	<0.0001
Most deprivation	6,025 (32.3)	1,680 (36.2)	
Mother's education at child birth			
Low (≤9 years)	2,322 (12.5)	711 (15.3)	
Medium (10-12 years)	8,077 (43.4)	2,162 (46.6)	<0.0001
High (>12 years)	8,216 (44.1)	1,764 (38.0)	
Father's education at child birth			
Low (≤9 years)	2,565 (13.9)	766 (16.7)	
Medium (10-12 years)	8,037 (43.6)	2,033 (44.3)	<0.0001
High (>12 years)	7,853 (42.5)	1,787 (39.0)	
Mother's employment during pregnancy			
Employed	13,991 (74.9)	3,369 (72.5)	
Unemployed with tasks	1,647 (8.8)	499 (10.8)	0.0001
Unemployed without tasks	3,032 (16.2)	776 (16.7)	
Father's employment during pregnancy			
Employed	16,057 (87.3)	3,848 (84.4)	
Unemployed with tasks	1,085 (5.9)	321 (7.0)	<0.0001
Unemployed without tasks	1,257 (6.8)	390 (8.6)	
Mother's marital status at child birth			
Married/Cohabiting	16,373 (87.9)	3,945 (85.1)	<0.0001
Single/Other situations	2,259 (12.1)	688 (14.9)	
Parental psychiatric history (F-diagnoses)			
Father diagnosed	1,963 (10.6)	531 (11.5)	<0.0001
Mother diagnosed	4,618 (24.9)	1,252 (27.2)	
Both parents diagnosed	1,806 (9.7)	606 (13.2)	

Table S6. Sensitivity analysis: Risk of ASD (overall, with and without ID) based on continuous pollutants' levels during different time windows including multiple births

Time windows and pollutants	Models A[†] OR (95% CI)	Models B[‡] OR (95% CI)	Models C[‖] OR (95% CI)
ASD overall (nr of cases/controls=5,298/18,822)			
Entire pregnancy			
NO_x (per 20μg/m^3 increase)	1.02 (0.95, 1.10)	1.02 (0.94, 1.10)	1.02 (0.94, 1.10)
PM_{10} (per 10μg/m^3 increase)	1.05 (0.91, 1.21)	1.02 (0.88, 1.17)	1.00 (0.87, 1.16)
First year of life			
NO_x (per 20μg/m^3 increase)	1.07 (0.98, 1.18)	1.06 (0.96, 1.16)	1.05 (0.96, 1.15)
PM_{10} (per 10μg/m^3 increase)	1.15 (0.98, 1.34)	1.08 (0.92, 1.27)	1.05 (0.89, 1.23)
ASD with ID (nr of cases/controls=954/18,822)			
Entire pregnancy			
NO_x (per 20μg/m^3 increase)	1.02 (0.86, 1.21)	1.05 (0.89, 1.25)	1.05 (0.89, 1.25)
PM_{10} (per 10μg/m^3 increase)	1.04 (0.76, 1.41)	1.07 (0.78, 1.47)	1.05 (0.76, 1.45)
First year of life			
NO_x (per 20μg/m^3 increase)	1.10 (0.91, 1.34)	1.09 (0.89, 1.33)	1.08 (0.88, 1.32)
PM_{10} (per 10μg/m^3 increase)	1.16 (0.83, 1.62)	1.11 (0.78, 1.57)	1.07 (0.75, 1.52)
ASD without ID (nr of cases/controls=4,344/18,822)			
Entire pregnancy			
NO_x (per 20μg/m^3 increase)	1.03 (0.95, 1.11)	1.01 (0.93, 1.10)	1.01 (0.93, 1.10)
PM_{10} (per 10μg/m^3 increase)	1.05 (0.91, 1.23)	1.00 (0.85, 1.17)	0.99 (0.84, 1.15)
First year of life			
NO_x (per 20μg/m^3 increase)	1.07 (0.97, 1.18)	1.05 (0.95, 1.16)	1.04 (0.94, 1.15)
PM_{10} (per 10μg/m^3 increase)	1.15 (0.97, 1.36)	1.07 (0.90, 1.28)	1.04 (0.87, 1.24)

[†] Models conditioned on calendar year and municipality of birth as well as adjusted for gender and birth month.

‡ Models conditioned on calendar year and municipality of birth as well as adjusted for gender, birth month, sib order, parents’ birth countries, mother's marital status, mother's education, father's education, mother's employment, father's employment, and disposable income within household.

‖ Models conditioned on calendar year and municipality of birth as well as adjusted for gender, birth month, sib order, parents’ birth countries, mother's marital status, mother's education, father's education, mother's employment, father's employment, disposable income within household, and neighborhood deprivation.

Table S7. Sensitivity analyses: Association between pollutants' levels during mother's pregnancy and child's first year of life and risk of childhood ASD overall by excluding children with certain conditions related to ASD.

	OR (95% CI)		
	Models A†	Models B‡	Models C‖
**Excluding children with ASD diagnoses before 2 years of age (no. of cases/controls=4,984/18,237)**			
Entire pregnancy			
NO_x (per 20μg/m^3 increase)	1.03 (0.95, 1.11)	1.02 (0.94, 1.10)	1.02 (0.94, 1.10)
PM_{10} (per 10μg/m^3 increase)	1.07 (0.92, 1.23)	1.02 (0.88, 1.18)	1.01 (0.87, 1.17)
First year of life			
NO_x (per 20μg/m^3 increase)	1.06 (0.97, 1.17)	1.04 (0.95, 1.15)	1.04 (0.94, 1.14)
PM_{10} (per 10μg/m^3 increase)	1.14 (0.97, 1.34)	1.07 (0.91, 1.26)	1.03 (0.88, 1.22)
**Excluding children born before 2003 (no. of cases/controls=1,210/7,172)**			
Entire pregnancy			
NO_x (per 20μg/m^3 increase)	0.87 (0.69, 1.09)	0.88 (0.70, 1.11)	0.88 (0.70, 1.11)
PM_{10} (per 10μg/m^3 increase)	0.99 (0.76, 1.30)	0.97 (0.74, 1.28)	0.96 (0.73, 1.26)
First year of life			
NO_x (per 20μg/m^3 increase)	0.89 (0.67, 1.18)	0.87 (0.65, 1.17)	0.86 (0.64, 1.15)
PM_{10} (per 10μg/m^3 increase)	1.13 (0.84, 1.53)	1.05 (0.77, 1.43)	1.01 (0.74, 1.38)
**Excluding children with congenital malformation (no. of cases/controls=4,862/17,707)**			
Entire pregnancy			
NO_x (per 20μg/m^3 increase)	1.03 (0.95, 1.11)	1.02 (0.94, 1.11)	1.02 (0.94, 1.11)
PM_{10} (per 10μg/m^3 increase)	1.06 (0.91, 1.23)	1.02 (0.88, 1.18)	1.00 (0.86, 1.17)
First year of life			
NO_x (per 20μg/m^3 increase)	1.06 (0.97, 1.17)	1.04 (0.95, 1.15)	1.03 (0.94, 1.14)
PM_{10} (per 10μg/m^3 increase)	1.13 (0.96, 1.33)	1.06 (0.90, 1.26)	1.03 (0.87, 1.22)
**Excluding children of either parent having any psychiatric disorders (no. of cases/controls= 2,018/10,379)**			
Entire pregnancy			
NO_x (per 20μg/m^3 increase)	1.08 (0.96, 1.21)	1.08 (0.96, 1.21)	1.08 (0.96, 1.21)
PM_{10} (per 10μg/m^3 increase)	1.26 (1.01, 1.55)	1.24 (0.99, 1.54)	1.22 (0.98, 1.52)

First year of life			
NO_x (per 20μg/m^3 increase)	1.08 (0.95, 1.24)	1.08 (0.94, 1.24)	1.07 (0.94, 1.23)
PM_{10} (per 10μg/m^3 increase)	1.22 (0.96, 1.55)	1.18 (0.93, 1.51)	1.15 (0.90, 1.48)
**Excluding children of mothers without pre-eclampsia (no. of cases/controls=4,776/17,421)**			
Entire pregnancy			
NO_x (per 20μg/m^3 increase)	0.87 (0.69, 1.09)	0.88 (0.70, 1.11)	0.88 (0.70, 1.11)
PM_{10} (per 10μg/m^3 increase)	0.99 (0.76, 1.30)	0.97 (0.74, 1.28)	0.96 (0.73, 1.26)
First year of life			
NO_x (per 20μg/m^3 increase)	0.89 (0.67, 1.18)	0.87 (0.65, 1.17)	0.86 (0.64, 1.15)
PM_{10} (per 10μg/m^3 increase)	1.13 (0.84, 1.53)	1.05 (0.77, 1.43)	1.01 (0.74, 1.38)
**Excluding children of mothers without pre-gestational and gestational diabetes (no. of cases/controls=5,037/18,072)**			
Entire pregnancy			
NO_x (per 20μg/m^3 increase)	1.02 (0.95, 1.11)	1.02 (0.94, 1.10)	1.02 (0.94, 1.10)
PM_{10} (per 10μg/m^3 increase)	1.04 (0.90, 1.21)	1.01 (0.87, 1.17)	0.99 (0.85, 1.15)
First year of life			
NO_x (per 20μg/m^3 increase)	1.06 (0.97, 1.17)	1.05 (0.95, 1.15)	1.04 (0.94, 1.14)
PM_{10} (per 10μg/m^3 increase)	1.12 (0.96, 1.32)	1.06 (0.90, 1.25)	1.02 (0.87, 1.20)
**Excluding children of mothers without placental abruption (no. of cases/controls=5,107/18,172)**			
Entire pregnancy			
NO_x (per 20μg/m^3 increase)	1.02 (0.95, 1.11)	1.02 (0.94, 1.10)	1.02 (0.94, 1.10)
PM_{10} (per 10μg/m^3 increase)	1.04 (0.90, 1.21)	1.01 (0.87, 1.17)	0.99 (0.85, 1.15)
First year of life			
NO_x (per 20μg/m^3 increase)	1.06 (0.97, 1.17)	1.05 (0.95, 1.15)	1.04 (0.94, 1.14)
PM_{10} (per 10μg/m^3 increase)	1.12 (0.96, 1.32)	1.06 (0.90, 1.25)	1.02 (0.87, 1.20)
**Excluding children of mothers without premature rupture of the membranes (no. of cases/controls=5,033/18,009)**			
Entire pregnancy			
NO_x (per 20μg/m^3 increase)	1.02 (0.95, 1.11)	1.02 (0.94, 1.10)	1.02 (0.94, 1.10)
PM_{10} (per 10μg/m^3 increase)	1.04 (0.90, 1.21)	1.01 (0.87, 1.17)	0.99 (0.85, 1.15)
First year of life			

NO_x (per 20μg/m^3 increase)	1.06 (0.97, 1.17)	1.05 (0.95, 1.15)	1.04 (0.94, 1.14)
PM_{10} (per 10μg/m^3 increase)	1.12 (0.96, 1.32)	1.06 (0.90, 1.25)	1.02 (0.87, 1.20)
**Excluding children of foreign-born parents[1] (no. of cases/controls=3,512/12,796)**			
Entire pregnancy			
NO_x (per 20μg/m^3 increase)	1.02 (0.94, 1.10)	1.01 (0.93, 1.10)	1.01 (0.93, 1.10)
PM_{10} (per 10μg/m^3 increase)	1.03 (0.88, 1.20)	0.99 (0.84, 1.15)	0.97 (0.83, 1.13)
First year of life			
NO_x (per 20μg/m^3 increase)	1.08 (0.98, 1.18)	1.06 (0.96, 1.16)	1.05 (0.95, 1.15)
PM_{10} (per 10μg/m^3 increase)	1.15 (0.97, 1.36)	1.07 (0.90, 1.26)	1.02 (0.86, 1.22)
**Excluding children with less than 37 weeks of gestation (no. of cases/controls=4,776/17,421)**			
Entire pregnancy			
NO_x (per 20μg/m^3 increase)	1.02 (0.94, 1.10)	1.01 (0.93, 1.09)	1.01 (0.93, 1.09)
PM_{10} (per 10μg/m^3 increase)	1.06 (0.91, 1.22)	1.02 (0.87, 1.18)	1.00 (0.86, 1.17)
First year of life			
NO_x (per 20μg/m^3 increase)	1.06 (0.96, 1.16)	1.04 (0.94, 1.14)	1.03 (0.94, 1.14)
PM_{10} (per 10μg/m^3 increase)	1.12 (0.95, 1.33)	1.06 (0.90, 1.26)	1.03 (0.87, 1.21)
**Excluding children with less than 2,500 g of birth weight (no. of cases/controls=4,840/17,671)**			
Entire pregnancy			
NO_x (per 20μg/m^3 increase)	1.03 (0.95, 1.12)	1.03 (0.95, 1.11)	1.03 (0.95, 1.11)
PM_{10} (per 10μg/m^3 increase)	1.07 (0.92, 1.24)	1.03 (0.89, 1.20)	1.02 (0.88, 1.19)
First year of life			
NO_x (per 20μg/m^3 increase)	1.05 (0.96, 1.16)	1.04 (0.94, 1.14)	1.03 (0.93, 1.13)
PM_{10} (per 10μg/m^3 increase)	1.12 (0.95, 1.32)	1.05 (0.89, 1.25)	1.02 (0.86, 1.21)

† Models conditioned on calendar year and municipality of birth as well as adjusted for gender and birth month.

‡ Models conditioned on calendar year and municipality of birth as well as adjusted for gender, birth month, sib order, parents' birth countries, mother's marital status, mother's education, father's education, mother's employment, father's employment, and disposable income within household.

‖ Models conditioned on calendar year and municipality of birth as well as adjusted for gender, birth month, sib order, parents' birth countries, mother's marital status, mother's education, father's education, mother's employment, father's employment, disposable income within household, and neighborhood deprivation.

[1] We did not adjust for parents' birth countries in the analyses of this subpopulation.

Table S8. Risk of ASD (overall, with and without ID) based on continuous pollutants' levels during different time windows without conditioning on municipality of birth

Time windows and pollutants	Models A[†]<br>OR (95% CI)	Models B[‡]<br>OR (95% CI)	Models C[ǁ]<br>OR (95% CI)
ASD overall (nr of cases/controls=5,136/18,237)			
Entire pregnancy			
NO_x (per 20µg/m^3 increase)	0.92 (0.87, 0.98)	0.92 (0.87, 0.98)	0.92 (0.87, 0.98)
PM_{10} (per 10µg/m^3 increase)	0.89 (0.81, 0.99)	0.87 (0.79, 0.97)	0.87 (0.78, 0.97)
First year of life			
NO_x (per 20µg/m^3 increase)	0.93 (0.87, 0.99)	0.91 (0.86, 0.98)	0.91 (0.85, 0.98)
PM_{10} (per 10µg/m^3 increase)	0.91 (0.82, 1.01)	0.88 (0.79, 0.98)	0.86 (0.77, 0.96)
ASD with ID (nr of cases/controls=913/118)			
Entire pregnancy			
NO_x (per 20µg/m^3 increase)	0.92 (0.82, 1.05)	1.01 (0.89, 1.15)	1.02 (0.90, 1.16)
PM_{10} (per 10µg/m^3 increase)	0.96 (0.78, 1.19)	1.08 (0.86, 1.36)	1.07 (0.85, 1.35)
First year of life			
NO_x (per 20µg/m^3 increase)	0.95 (0.83, 1.08)	1.02 (0.89, 1.17)	1.02 (0.89, 1.17)
PM_{10} (per 10µg/m^3 increase)	1.02 (0.82, 1.26)	1.09 (0.87, 1.38)	1.08 (0.85, 1.37)
ASD without ID (nr of cases/controls=4,223/18,119)			
Entire pregnancy			
NO_x (per 20µg/m^3 increase)	0.92 (0.87, 0.98)	0.90 (0.84, 0.96)	0.90 (0.85, 0.96)
PM_{10} (per 10µg/m^3 increase)	0.88 (0.79, 0.98)	0.83 (0.74, 0.94)	0.83 (0.73, 0.93)
First year of life			
NO_x (per 20µg/m^3 increase)	0.92 (0.86, 0.99)	0.89 (0.83, 0.96)	0.89 (0.83, 0.96)
PM_{10} (per 10µg/m^3 increase)	0.89 (0.79, 1.00)	0.84 (0.74, 0.94)	0.82 (0.73, 0.93)

[†] Models conditioned on calendar year of birth, as well as adjusted for gender and birth month.

‡ Models conditioned on calendar year of birth, as well as adjusted for gender, birth month, sib order, parents' birth countries, mother's marital status, mother's education, father's education, mother's employment, father's employment, and disposable income within household.

‖ Models conditioned on calendar year of birth, as well as adjusted for gender, birth month, sib order, parents' birth countries, mother's marital status, mother's education, father's education, mother's employment, father's employment, disposable income within household, and neighborhood deprivation.
